# Task relevance modulates the cortical representation of feature conjunctions in the target template

**DOI:** 10.1038/s41598-017-04123-8

**Published:** 2017-07-03

**Authors:** Reshanne R. Reeder, Michael Hanke, Stefan Pollmann

**Affiliations:** 1 Department of Experimental Psychology, Institute of Psychology II, Otto-von-Guericke University, Magdeburg, Germany; 2 Psychoinformatics Lab, Institute of Psychology II, Otto-von-Guericke University, Magdeburg, Germany; 3 Center for Behavioral Brain Sciences, Magdeburg, Germany

## Abstract

Little is known about the cortical regions involved in representing task-related content in preparation for visual task performance. Here we used representational similarity analysis (RSA) to investigate the BOLD response pattern similarity between task relevant and task irrelevant feature dimensions during conjunction viewing and target template maintenance prior to visual search. Subjects were cued to search for a spatial frequency (SF) or orientation of a Gabor grating and we measured BOLD signal during cue and delay periods before the onset of a search display. RSA of delay period activity revealed that widespread regions in frontal, posterior parietal, and occipitotemporal cortices showed general representational differences between task relevant and task irrelevant dimensions (e.g., orientation vs. SF). In contrast, RSA of cue period activity revealed sensory-related representational differences between cue images (regardless of task) at the occipital pole and additionally in the frontal pole. Our data show that task and sensory information are represented differently during viewing and during target template maintenance, and that task relevance modulates the representation of visual information across the cortex.

## Introduction

When you look for a banana in a fruit basket, you can guide your search towards distinct features (e.g., color, curvature) while ignoring other features or feature dimensions. Such goal-directed attention requires a large network of brain regions interacting in different ways. Classically, a frontoparietal network (superior and middle frontal gyri to intraparietal sulcus) is thought to be responsible for directing spatial attention control signals to visual cortex^1, 2^ even prior to visual stimulation^3^, suggesting these regions contribute to preparatory attention. There is evidence this network is also involved in biasing attention to task relevant features, both during viewing^4, 5^ and in preparation for viewing expected features^6^. Anterior regions of cortex are thought to be involved in sending attention control signals to more posterior sensory regions, where stimulus contents are thought to be represented in working memory^7, 8^. There is also evidence that stimuli that are currently not task relevant are suppressed in sensory cortices^9^.

Studies have typically analyzed changes in regional blood-oxygen-level-dependent (BOLD) amplitudes to infer attention modulation based on task relevance, but have more recently adopted computational methods to investigate voxel-level activity patterns associated with different experimental conditions of interest; this has enabled us to analyze the representational content of brain activation^10^. These methods have particular use in investigating feature representation in working memory in the absence of visual stimulation. Maintaining task relevant information in working memory corresponds to a theoretical “target template”^1, 11^. The pre-activation of target-specific patterns has been observed throughout the ventral visual stream: from striate visual cortex^12, 13^ to extrastriate areas^12, 14^ and lateral occipital cortex (LOC^15–17^), representing various stimulus-related information such as precise visual features (e.g., oriented bars), feature dimensions (e.g., orientation or spatial frequency; SF), objects, and categories.

There are currently mixed findings about the extent to which frontoparietal attention control regions^18, 19^ are involved in preparation for target detection. Several studies using pattern classification methods have found evidence that posterior parietal cortex is involved in both sending control signals to ventral visual areas and representing the contents of working memory^20, 21^. Some results maintain that frontal regions are content-free^20^, but a recent study reconstructed working memory content activated in frontal cortex using encoding model techniques^22^.

**Figure 1:**
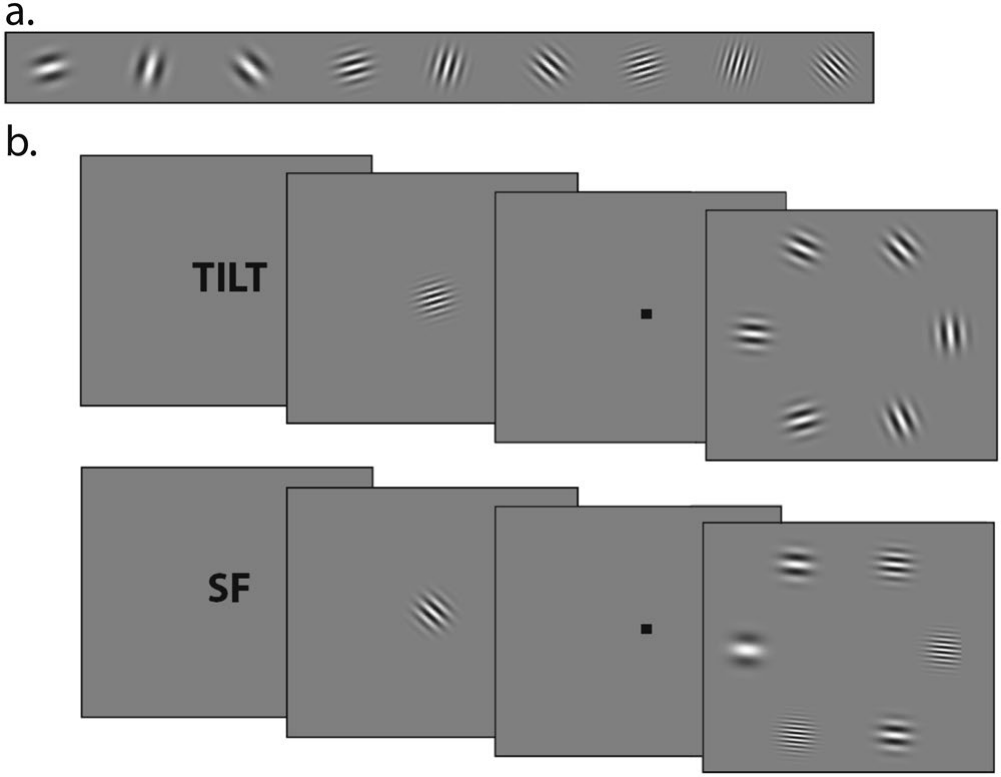
(**a**) The 9 Gabor images used as cues in the experiment. (**b**) The experimental procedure for the two search tasks.

In the current study, we used representational similarity analysis (RSA^23, 24^) to investigate the similarity of activity patterns elicited by task relevant and task irrelevant feature dimensions (e.g., a target defined by its orientation vs. SF) during viewing and target template maintenance prior to visual search. During fMRI, subjects were instructed at the beginning of each trial to search for the orientation or SF of a cue Gabor grating. Cues were followed by a fixation period (delay), after which a search display appeared containing six Gabor gratings, one of which contained the cued feature. In the SF search task, the target appeared with a different orientation than the cue, and in the orientation search task, the target appeared with a different SF than the cue (see Fig. 1). Subjects were therefore motivated to prepare only for the current task relevant information and to ignore the task irrelevant information provided by the cue. Our time periods of interest were the cue period (i.e., the stimulus processing period) and the delay period prior to the onset of the search display (i.e., the target template maintenance period). The durations of both time periods were jittered orthogonally so we could analyze their BOLD responses separately.

**Figure 2:**
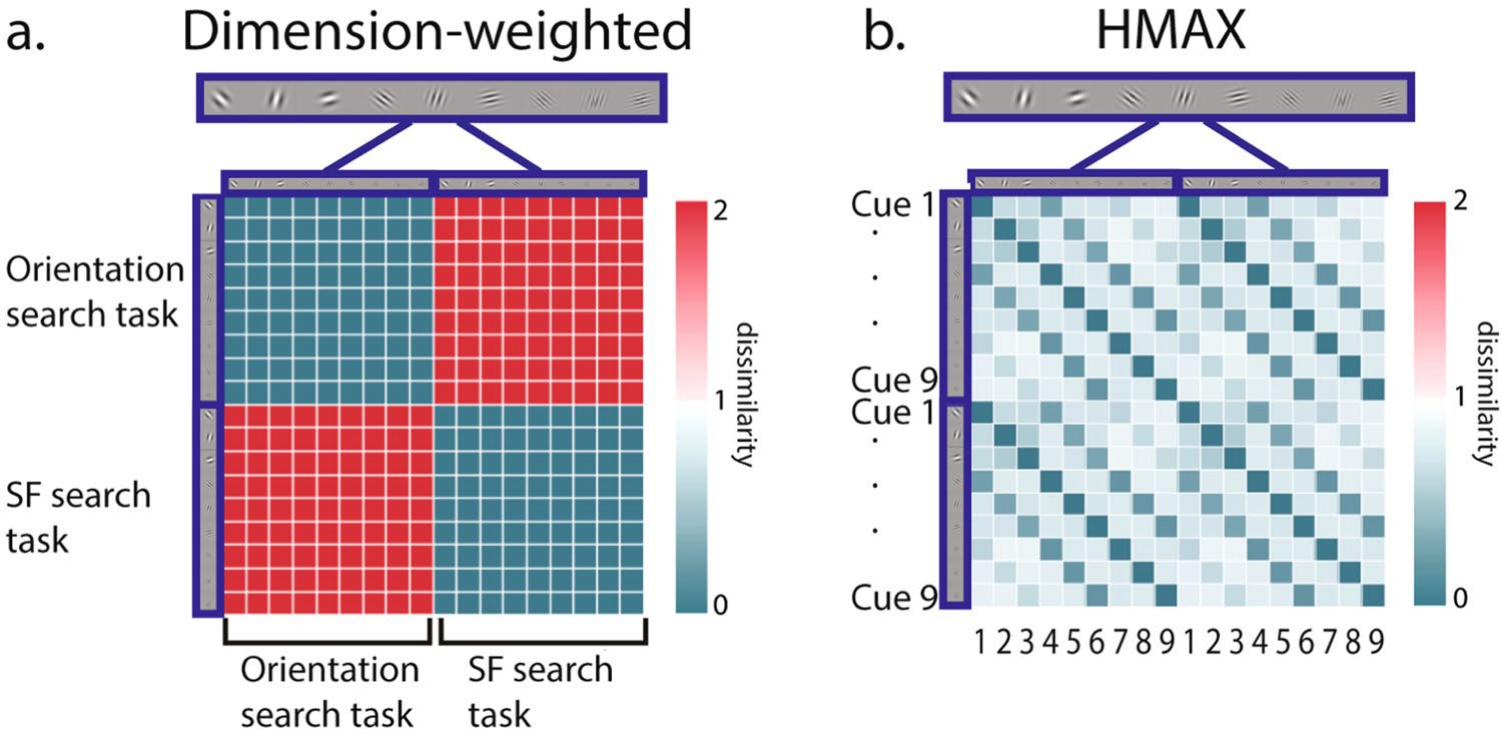
(**a**) The dimension-weighted model RDM, with the cue images represented maximally similarly within the task relevant feature dimension and maximally differently from the task irrelevant feature dimension. (**b**) The HMAX C1 model RDM, representing correlations between simulated visual activity in response to the 9 viewed cue images in the experiment.

It has been long known that attention weighting of a target-defining feature dimension can facilitate visual search^25, 26^. Therefore, we investigated where in the brain task relevant feature dimensions are represented as part of a general attention mechanism. Based on the working memory literature, as well as on previous neuroimaging studies of dimension-specific visual search^27^, we expected the task relevant feature dimension to be represented maximally differently from the task irrelevant feature dimension (see Fig. 2a) in classical attention regions extending from the frontal eye fields to posterior parietal cortex along the intraparietal sulcus.

To investigate the representation of purely sensory-driven information (i.e., the different feature conjunctions presented as cues unconstrained by task demands), we used a model of early sensory processing^23, 28^ to find brain regions that represent feature conjunctions as a unique pattern, regardless of search task performed (see Fig. 2b). Of particular interest was the question if left frontopolar cortex, an area had been observed to be activated during visual dimension changes^27^ would represent the task-relevant feature dimension or rather stimulus features independent of task relevance. The former would signal the result of a dimension change whereas the latter would indicate that left frontopolar cortex receives information about attended as well as not attended features, a precondition for detecting changes in the unattended dimension that may require a re-weighting of dimensional attention^27^.

## Materials and Methods

### Subjects

Three subjects were excluded due to low average accuracy on the search tasks (<70%). Remaining subjects were 11 students and researchers (3 female) recruited from the Universitätsklinikum fMRI subject pool in Magdeburg, Germany (age range = 25–35 years, mean age = 30.1 years). Subjects were all right-handed native German speakers and had participated in previous fMRI experiments unrelated to the current study. Subjects had normal or corrected-to-normal vision and received a monetary reimbursement for their participation. Prior to experimentation, subjects completed an fMRI screening questionnaire, received details about the experiment, and provided written, informed consent to take part in the experiment. These measures were approved by the research ethics committee of Otto-von-Guericke University Magdeburg in accordance with the approved guidelines.

### Stimuli

Stimuli were presented via a D-ILA projector to a back-projection screen placed in the bore of the scanner behind the participant’s head. Subjects viewed the screen through a mirror mounted on top of the head coil with 60 Hz refresh frequency. The experiment was coded in Python and implemented with the PsychoPy psychophysics toolbox^29^ version 1.82.01 on a Debian operating system using the NeuroDebian^30^ software platform.

Stimuli were Gabor gratings generated in PsychoPy with the function “GratingStim”, all with a sinusoidal texture and Gaussian mask (See Fig. 1a). Cue Gabors were a constant 5 degrees of visual angle in diameter. The cue Gabor could appear at one of three possible orientations (15, 75, or 135 degrees) and one of three possible SFs (1, 2, or 3 cycles per degree of visual angle). A search array was composed of 6 Gabors equally spaced in a circle with a radius of 8.37 degrees of visual angle. Gabors in the search display could appear at one of 9 possible orientations (each of the three cued orientations plus 35, 55, 95, 115, 155, 175) and one of 8 possible SFs (each of the three cued SFs plus 0.7, 1.3, 1.5, 3.8, or 4.3 cycles per degree of visual angle). These values were chosen to control for task difficulty behaviorally, but were not important for the fMRI analyses (only the BOLD responses in the cue period and delay period prior to the onset of the search display were analyzed).

### Experimental Procedure

The experiment was a visual search task in which subjects were required to respond whether a cued feature appeared on the left or right side of a search display (See Fig. 1b). Before the first experimental block, subjects completed practice trials to ensure they understood task instructions. At the beginning of each experimental block, an instruction message appeared that read: “Wenn die vorher gezeigte Eigenschaft auf der linken Seite erscheint, druecken Sie die 1. Wenn die vorher gezeigte Eigenschaft auf der rechten Seite erscheint, druecken Sie die 2” (If you see the cued feature on the right, press 1. If you see the cued feature on the left, press 2). All text appeared black against a gray screen and was presented at a height of 1 degree of visual angle. Following the block instructions, an fMRI trigger initiated the trial sequence.

Each trial started with an instruction screen for 1 second (s), prompting subjects to attend to a particular feature dimension of the cue: “TILT” (i.e., orientation), “SF”, or “EXACT IMAGE” (exact image trials were not analyzed in the current experiment). There were 9 trials for each task instruction within a block, with each task instruction presented in a pseudo-randomized, counterbalanced order using De Bruijn sequences for each block^31^.

Following the task instructions, a cue Gabor appeared for a duration determined by a logarithmic distribution so subjects could not predict cue offsets. The distribution of cue durations was restricted to 9 values between 1–2.5 s, which were each repeated 3 times per block of trials (total of 27 trials). The cue Gabor appeared in the center of the screen with a pseudo-randomly selected orientation and SF. Each of the 9 possible orientation/SF combinations appeared three times within a block – once for each task instruction – but the precise order of cues was shuffled every block.

After the cue, a central fixation point (a black visual grating stimulus generated in PsychoPy with a SF of 0 and size of 0.5 degrees of visual angle) was presented on screen for an inter-stimulus-interval determined by a logarithmic distribution so subjects could not predict fixation offsets or search display onsets. The distribution of fixation durations was restricted to 9 values between 2.5–6.5 s, which were each repeated 3 times per block of trials (total of 27 trials). These values were chosen independently of cue duration to maximally separate BOLD signal elicited during the cue period and delay period.

After fixation, a search display appeared for 4 s. If the task was to search for SF, all 6 Gabors shared the same orientation (randomly selected from the 6 orientations that were never the cued orientation) and all 6 Gabors appeared with different SFs (one of which was shared by the cue). If the task was to search for orientation, all 6 Gabors shared the same SF (randomly selected from the 5 SFs that were never the cued spatial frequency), and all 6 Gabors appeared with different orientations (one of which was shared by the cue). Gabors in a given search display were separated by 20 degrees, which removed a ceiling effect behaviorally (which possibly could have affected the precision of the target template).

If the task was to search for an exact image, 2 Gabors in the search display shared the same orientation as the cue (with different SFs), 2 Gabors shared the same SF (with different orientations), 1 Gabor appeared with a unique SF and orientation (different from all other Gabors in the display, to keep the number of Gabors with the same SF or same orientation as the cue consistent), and 1 Gabor appeared with the same SF and orientation as the cue. Exact image search trials were recorded for a separate study and were not included in the analyses of the current study. All models, analyses, and results of the current study are based on responses in the SF search and Orientation search tasks only.

Subjects were instructed to make a “left” or “right” response as fast and accurately as possible following the onset of the search display. Responses did not terminate a trial. Each block lasted 5 minutes 22 s. All subjects performed 8 blocks of trials, with approximately 45 minutes total experiment time with 72 trials per search task.

### fMRI data acquisition

Subjects were scanned on a 3 Tesla MAGNETOM Prisma (Siemens) at the Universitätsklinikum in Magdeburg, Germany. fMRI data were collected using a gradient-echo echo-planar imaging (EPI) sequence (TR, 2000 ms; TE, 30 ms; flip angle, 90°; matrix size, 80 × 80; FOV, 240 mm; 36 slices with interleaved acquisition; 3 × 3 × 3 mm voxels; 0.3 mm interslice gap). Each scanning session lasted 316 s. We also acquired structural data for each subject using a T1-weighted MPRAGE sequence (TR 2.5 s; TE 2.82 ms; TI 1.1 s; flip angle 7°; 1 mm isotropic resolution).

### fMRI preprocessing

fMRI data preprocessing was carried out in FSL version 5.0.8^32^. For each subject, we registered functional images to their respective T1-weighted anatomical space and then to Montreal Neurological Institute (MNI) space using FSL’s FLIRT linear registration tool^33^. Specifically, functional images were first registered to the middle-timepoint functional image for each run, which were then registered to the brain-extracted T1-weighted image^34^, and finally to the MNI template. For univariate analyses, these data were then smoothed with a 6 mm FWHM Gaussian kernel prior to analysis.

One experimental model was analyzed for each subject in the univariate analyses, modeling the cue period and delay period so that these time periods could be directly compared. The cue period and delay period were modeled separately for the searchlight analyses because a model that takes time period as an added parameter would have to include predictions about how each condition in the cue period would fit to each condition in the delay period (and vice versa), and we did not want to make such assumptions. For the model-driven searchlights, we smoothed the data with a 4 mm FWHM Gaussian kernel (to keep fine spatial patterns intact) using the Nilearn Python package^35^, and fitted an event-related hemodynamic response function (HRF) model to the smoothed dataset using NiPy^36^. Regressors for the HRF model were determined separately for the cue period and delay period based on precise onsets and offsets of each time period on a trial-by-trial basis, and were modeled for 18 separate experimental conditions (see “Model-driven analyses” in the “Analyses” section below). Finally, we voxelwise Z-scored across all HRF parameter images prior to group level analyses.

### Analyses

#### Univariate analyses

Group level univariate analyses were conducted using FSL’s FEAT function^32^ on functional data registered to MNI space. BOLD responses were analyzed for the cue period and the delay period in a single model, and regressors were created based on precise timing of onsets and offsets of these periods on a trial-by-trial basis. We modeled the BOLD signal with a double-gamma HRF and we corrected for multiple comparisons using cluster-based thresholding on group level data with FSL’s FEAT function for higher-level analyses. The cluster-forming Z-threshold was set to 2.3 and all univariate results shown in the current paper are above this threshold. All clusters reported in the paper have a significance threshold of *p* < 0.01.

**Figure 3:**
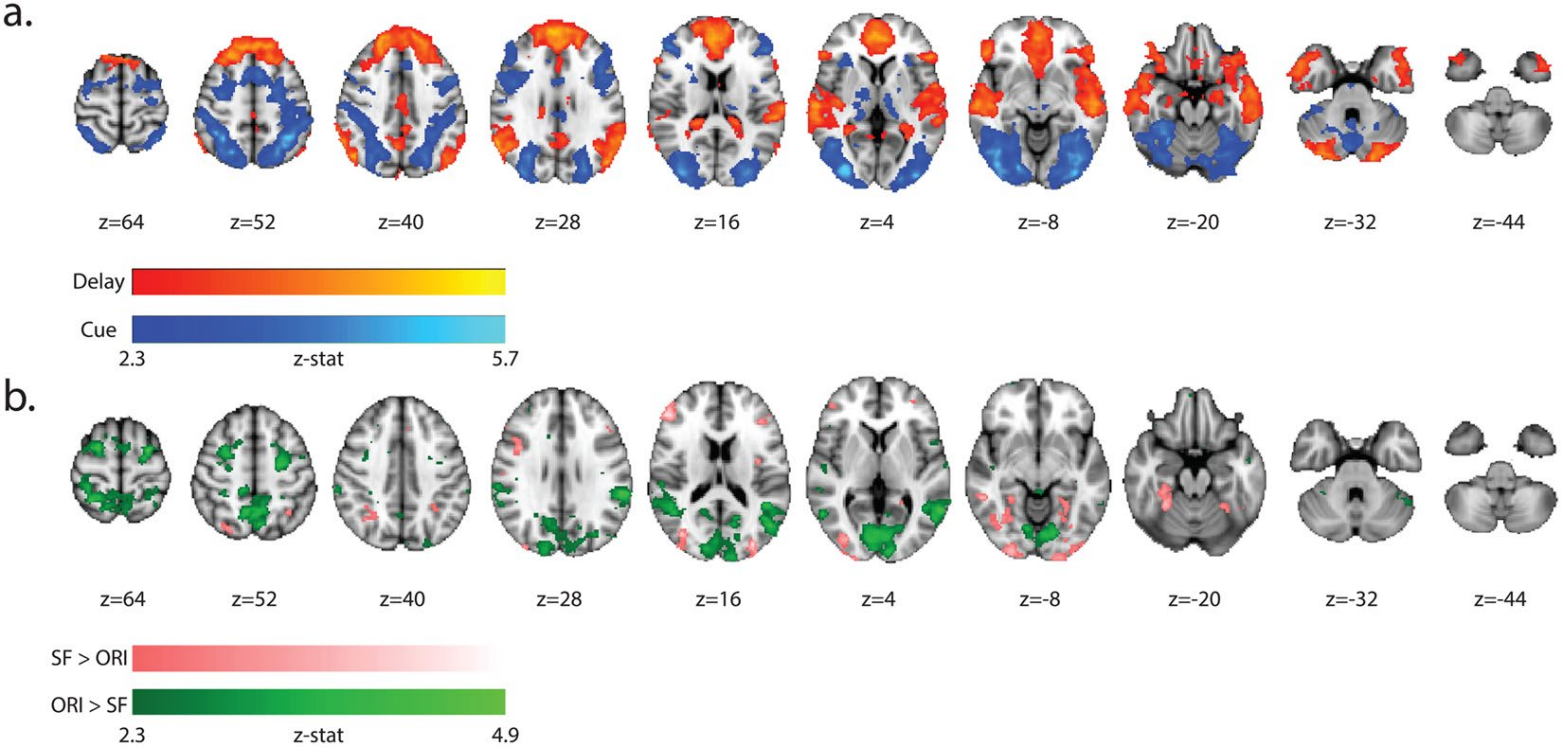
All colored areas represent z-values above 2.3 at a significance threshold of *p*_corrected_ < 0.01. (**a**) Axial slices in MNI space depicting univariate contrasts for cue period > delay period BOLD in dark-to-light blue and delay period > cue period BOLD in red-to-yellow. (**b**) Axial slices in MNI space depicting univariate contrasts for cue period SF search task > Orientation search task in dark-to-light pink and cue period Orientation search task > SF search task in dark-to-light green.

Task contrasts of interest were cue period > delay period BOLD and delay period > cue period BOLD. These contrasts were performed to determine whether the time periods of interest were functionally separable and whether we could replicate long-standing assumptions that delay period signals are dominant in frontal regions and stimulus processing signals are dominant in posterior regions^37^. We further performed the univariate contrasts Orientation search task > SF search task and SF search task > Orientation search task within each time period separately, to evaluate the BOLD responses attributed to general task differences.

#### Representational Similarity Analysis (RSA)

RSA^24^ is a pattern analysis technique that can be used to determine the similarity between patterns of activity for different conditions of interest. Each condition of interest is compared to each other in a representational dissimilarity matrix (RDM) that encodes the distance between all pairwise combinations of conditions. Correlation distance was used as the representational distance (RD) metric of choice in this study, calculated as 1 minus the Pearson correlation between conditions, so if a RD value is low for a pair of conditions (i.e., close to 0), then correlations are high. RD values are on a scale from 0–2, with 0 being perfectly correlated, 1 being not correlated, and 2 being perfectly anti-correlated. In our study, correlations between beta weights were calculated for 18 conditions of interest (9 cue images × 2 search tasks) contributing to a model. All RSA results report the Fisher z-transformation values on the Pearson correlation distance between conditions.

To investigate the influence of task relevance on feature dimension representation, we created a dimension-weighted model RDM that followed specific hypotheses: that the task relevant feature dimension would be represented maximally differently from the task irrelevant feature dimension, and that stimuli would be represented maximally similarly within the task relevant feature dimension (see Fig. 2a). The latter followed from the assumption that attention to a feature dimension leads to enhanced processing of all features on the relevant dimension relative to features on the irrelevant dimension.

To investigate sensory feature representation, we generated an HMAX C1 RDM to model the representational information provided by the cue images unconstrained by task demands. HMAX is a model that simulates responses to visual input throughout the brain and the C1 layer of the model simulates complex cell responses in visual cortex. To generate simulated responses, we input each cue image into the model, which delivered an output of expected beta values in response to viewing the images. We then created a matrix that gauged the correlation between simulated beta values for each cue image, resulting in the model shown in Fig. 2b.

To determine clusters with significant group level correlations between observed RDMs and the models we employed a bootstrapped permutation analysis^38^ implemented in PyMVPA. For each subject 150 additional searchlight correlation maps were computed using newly permuted RDM labels for every iteration^24^. By randomly selecting one map per subject and averaging (Fisher z-transformed) correlation maps across all subjects, 10000 bootstrap samples were generated. The voxel-wise cluster forming threshold was determined by inspecting the distribution of z-values estimated via the bootstrap at each voxel, choosing the threshold to match a probability of *p*_corrected_ < 0.01 for observing a score of this magnitude or higher. Subsequently, the distribution of cluster sizes was determined from the thresholded bootstrap correlation maps. The reported results reflect super-threshold cluster with sizes corresponding to *p*_corrected_ < 0.01 (FWE-corrected). In addition to size and probability, we report mean and maximum Fisher z-transformed Pearson correlation between the models and observed RD patterns for each cluster. It should be noted that the reported probabilities do not correspond to individual correlation magnitudes, but represent cluster-level statistics.

The HMAX C1 model did not reveal any significant clusters of activity when the cluster-forming threshold was *p*_corrected_ < 0.01; therefore, for this model, the cluster-forming threshold was raised to *p*_corrected_ < 0.05. We performed the thresholding procedure for correlation clusters at the group level for the cue period and delay period separately for each model.

## Results

### Behavioral results

We analyzed each subject’s accuracy, reaction time (RT; only correct trials included), and Inverse Efficiency Score (IES; an accuracy-weighted RT value (RT/accuracy) that controls for speed-accuracy tradeoffs) for each of the search tasks. Behavioral results (accuracy, RT, IES) were submitted to separate paired-samples t-tests (SF search vs. Orientation search).

There were no significant differences in accuracy between the two task conditions, *t*(10) = −1.05, *p* = 0.32 (standard error of the mean (SEM) is reported here; SF search task: 81.4% ± 2.2%, Orientation search task: 85.3% ± 2.6%). There were significant differences in RT between the two tasks, *t*(10) = −3.87, *p* = 0.003 (SF search task: 1.97 s ± 0.11 s, Orientation search task: 2.20 s ± 0.10 s). However, when both speed and accuracy were taken into account (using IES as the measure of performance), the differences between conditions vanished: *t*(10) = −1.12, *p* = 0.288 (SF search task: 2.44 s ± 0.16 s, Orientation search task: 2.60 s ± 0.13 s). These results suggest the search tasks were well matched for difficulty when controlling for speed-accuracy tradeoffs.

### Imaging results

#### Univariate results

There were 2 large clusters with significant cue period > delay period BOLD signal amplitudes. The first cluster extended from the occipital pole ventrally to bilateral occipital fusiform gyrus, and LOC, and dorsally along the full extent of the bilateral intraoccipital/intraparietal sulcus; the second cluster was observed bilaterally at the junction of superior precentral sulcus and superior frontal sulcus, i.e., the location of the frontal eye fields, extending medially to the location of the supplementary eye fields and laterally to middle frontal gyrus. There was a third small cluster in the rostral part of the parahippocampal gyrus, extending into the left thalamus (see Fig. 3a). There were 3 clusters with significant delay period > cue period BOLD amplitudes: one extending through large regions of the frontal lobes, including frontal pole, bilateral superior frontal gyrus, orbitofrontal gyrus, and anterior cingulate cortex; one in angular gyrus extending into anterior temporal cortex; and one extending from the posterior portion of anterior cingulate cortex to posterior cingulate gyrus, precuneus and retrosplenial cortex; see Fig. 3a).

There were no significant clusters in any region of the brain for either of the further delay-period univariate contrasts performed (Orientation search task > SF search task, SF search task > Orientation search task). The absence of a significant difference in BOLD signal amplitudes between the single-feature search tasks in the delay period assures that representational differences found with RSA for this time period cannot simply be attributed to regional blood flow changes due to general task differences, such as task difficulty. Further cue period univariate contrasts revealed regional blood flow differences between attending to viewed orientations vs. SFs (see Fig. 3b). The Orientation search task > SF search task contrast revealed 6 significant clusters: one extending bilaterally through the lingual gyrus and calcarine cortex extending into supracalcarine cortex; one in right superior parietal lobule and precuneus; bilaterally in left and right middle temporal gyrus; and bilaterally in posterior left and right superior frontal gyrus, including the frontal eye fields. The SF search task > Orientation search task contrast revealed 2 significant clusters bilaterally in fusiform gyri, extending into the occipital pole region.

#### RSA results

**Figure 4:**
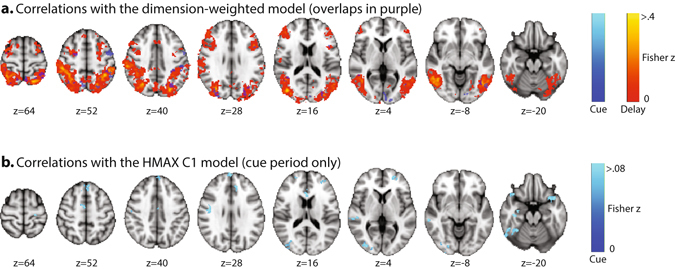
(**a**) Axial slices in MNI space depicting the Fisher z score distributions within clusters that showed significant correlations with the dimension-weighted model RDM in the cue period (dark-to-light blue) and delay period (red-to-yellow) at a significance threshold of *p*_corrected_ < 0.01. (**b**) Axial slices in MNI space depicting the Fisher z score distribution within clusters that showed significant correlations with the HMAX C1 model RDM in the cue period (dark-to-light blue) at a significance threshold of *p*_corrected_ < 0.05.

Figure 4a shows Fisher z-transformed correlations with the dimension-weighted model for the cue period (dark-to-light blue) and the delay period (red-to-yellow) with cue period activity depicted as a transparent overlay on delay period activity. There were 8 clusters in the cue period that showed significant correlated activity patterns with the model. The largest clusters were in left superior LOC and left superior parietal lobule. Smaller clusters appeared in a region overlapping precentral gyrus, left inferior frontal gyrus pars opercularis, and left middle frontal gyrus; left occipital pole overlapping the lingual gyrus, calcarine and supracalcarine cortices; right superior LOC extending into precuneus; right superior LOC extending into occipital pole; and right middle temporal gyrus extending into angular gyrus. The delay period showed much more extensive activity patterns compared to the cue period. There were large bilateral clusters covering extensive posterior regions running ventrally from the occipital pole through inferior LOC (the location of the cluster maximum) into middle temporal gyrus and inferior temporal gyrus, and dorsally along the whole extent of the intraparietal sulcus. Smaller clusters of significant correlations with the model also extended anteriorly from the junction of superior precentral sulcus and superior frontal sulcus corresponding to the frontal eye fields, in medial frontal cortex corresponding to the supplementary eye fields, and in left prefrontal cortex reaching into rostral frontal cortex. Comparing the cue period to the delay period, many of the cue period clusters overlapped those activated during the delay period (see Fig. 4a), with the exception of 2 clusters unique to the cue period found in left precentral gyrus overlapping inferior frontal gyrus pars opercularis and middle frontal gyrus; and left occipital pole overlapping lingual gyrus and calcarine cortex. See Tables 1 and 2 for a full breakdown of significant searchlight clusters for the cue period and delay period.

**Table 1:** Clusters from the cue period that showed significant correlated activity patterns with the dimension-weighted model.

Cluster #	Size (voxels)	Max Fisher z	Max location (MNI)	Mean Fisher z	Std	Center of mass (MNI)	*p*corr	Structure (Harvard-Oxford Cortical Structural Atlas)
1	711	0.1984	−30, −84, 32	0.0980	0.0210	−24.8, −75.9, 36.3	0.0003	Left Superior Lateral Occipital Cortex
2	641	0.1723	−30, −50, 72	0.1029	0.0185	−38, −45.7, 57.2	0.0003	Left Superior Parietal Lobule
3	298	0.1641	−54, 2, 34	0.1029	0.0176	−48.9, 0.6, 43.9	0.0009	Left Precentral Gyrus overlapping Inferior Frontal Gyrus pars opercularis, Middle Frontal Gyrus
4	261	0.1453	−20, −98, 8	0.0955	0.0163	−9, −91.3, 2.9	0.0009	Left Occipital Pole overlapping Lingual Gyrus, Calcarine Cortex
5	161	0.1576	30, −66, 58	0.1012	0.0180	31.3, −64.1, 55.9	0.0027	Right Superior Lateral Occipital Cortex overlapping Precuneous
6	151	0.1535	34, −80, 30	0.1021	0.0160	30.5, −83.9, 28	0.0027	Right Superior Lateral Occipital Cortex overlapping Occipital Pole
7	139	0.1657	12, −68, 46	0.1045	0.0194	10.4, −65.4, 53.3	0.0028	Right Precuneous overlapping Superior Lateral Occipital Cortex
8	105	0.1715	50, −48, 8	0.1045	0.0175	46.7, −51, 9.7	0.0051	Right Middle Temporal Gyrus temporooccipital part extending into Angular Gyrus

**Table 2:** Clusters from the delay period that showed significant correlated activity patterns with the dimension-weighted model.

Cluster #	Size (voxels)	Max Fisher z	Max location (MNI)	Mean Fisher z	Std	Center of mass (MNI)	*p*corr	Structure (Harvard-Oxford Cortical Structural Atlas)
1	27895	0.4164	46, −62, −2	0.1274	0.0520	4.5, −59.5, 30.1	0.0001	Right Inferior Lateral Occipital Cortex extending ventrally from Occipital Pole to bilateral Inferior Temporal Gyrus and Middle Temporal Gyrus. Extending dorsally to bilateral Superior Lateral Occipital Cortex, Angular Gyrus, and into Superior Parietal Lobule
2	3915	0.3070	56, 10, 24	0.1078	0.0314	42.4, 18.1, 34.7	0.0001	Right Precentral Gyrus extending into Inferior Frontal Gyrus pars opercularis, Middle Frontal Gyrus, Superior Frontal Gyrus, and Frontal Pole
3	2582	0.2621	−44, 14, 26	0.1021	0.0296	−38.8, 5, 33.6	0.0001	Left Inferior Frontal Gyrus pars opercularis and pars triangularis extending into Middle Frontal Gyrus, Precentral Gyrus, and Frontal Pole
4	222	0.1421	0, −38, 36	0.0898	0.0150	3.2, −35.3, 38.2	0.0031	Posterior Cingulate Gyrus

**Figure 5:**
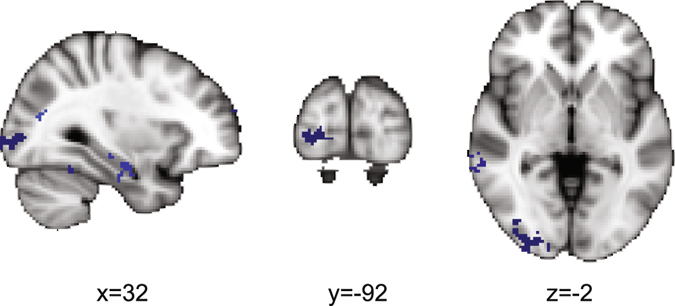
Sagittal, coronal, and axial slices in MNI space of the posterior cluster extending from the right LOC to the right occipital pole at a significance threshold of *p*_corrected_ < 0.05.

Figure 4b shows Fisher z-transformed correlations with the HMAX C1 model of sensory processing. There were 13 significant clusters that emerged in the cue period. The largest cluster appeared in a region of right inferior LOC at the occipital pole (shown in more detail in Fig. 5). Additional clusters occurred in left frontal regions including the frontal pole, right temporooccipital regions, and bilateral temporal pole (see Table 3 for a breakdown of all reported clusters for this model). Of note, a significant correlation was observed in left frontopolar cortex, at the junction of the intermediate frontal sulcus and the frontomarginal sulcus, an area that has previously been observed in cross-dimensional attention weighting^39^.

**Table 3:** Clusters from the cue period that showed significant correlated activity patterns with the HMAX C1 model.

Cluster #	Size (voxels)	Max Fisher z	Max location (MNI)	Mean Fisher z	Std	Center of mass (MNI)	*p*corr	Structure (Harvard-Oxford Cortical Structural Atlas)
1	213	0.1029	32, −90, 0	0.0645	0.0104	30.4, −88.6, −1.5	0.0393	Right Inferior Lateral Occipital Cortex overlapping Occipital Pole
2	192	0.1029	0, 60, 30	0.0694	0.0094	8.8, 60.4, 27.3	0.0393	Left Frontal Pole overlapping with Superior Frontal Gyrus
3	174	0.1070	34, −50, −20	0.0678	0.0094	47.8, −53.8, −20.1	0.0393	Right Temporal Occipital Fusiform Cortex overlapping Inferior Temporal Gyrus
4	172	0.0996	54, −28, 6	0.0678	0.0097	61.8, −33.5, −3.6	0.0393	Right Posterior Superior Temporal Gyrus overlapping Posterior Middle Temporal Gyrus
5	172	0.1094	46, −10, 28	0.0678	0.0111	46.5, −6.1, 31.1	0.0393	Right Post− and Precentral Gyrus
6	163	0.1339	−40, 14, −22	0.0784	0.0156	−38.6, 15.7, −22.3	0.0393	Left Temporal Pole extending into Frontal Orbital Cortex
7	155	0.1094	−4, 36, 58	0.0702	0.0113	−1.2, 34.1, 55.2	0.0393	Left Superior Frontal Gyrus extending into right Superior Frontal Gyrus
8	133	0.0939	−2, 28, 22	0.0719	0.0096	−3, 28, 21.9	0.044	Left Anterior Cingulate Gyrus
9	127	0.0972	−32, 46, 18	0.0686	0.0085	−29.3, 49.7, 10.1	0.044	Left Frontal Pole
10	127	0.1053	46, 26, −26	0.0710	0.0100	43.8, 20.1, −24.4	0.044	Right Temporal Pole
11	120	0.1176	−20, −26, 70	0.0727	0.0122	−22.6, −25.4, 70.7	0.0461	Left Precentral Gyrus
12	118	0.1429	8, −6, 48	0.0727	0.0147	5.2, −6.5, 49	0.0461	Right Juxtapositional Lobule Cortex extending into Anterior Cingulate Gyrus
13	115	0.0849	36, −80, 18	0.0621	0.0071	38.5, −79.8, 16.4	0.0461	Right Superior Lateral Occipital Cortex extending into Occipital Pole

## Discussion

How does the brain represent a target in preparation for visual search? Here we investigated the representation of task relevant feature dimensions as attention templates, i.e., working memory representations of targets in an upcoming search task. Previous work has consistently found dorsal and ventral attention network involvement in crossdimensional feature conjunction search^40–42^, attention shifts between dimensions, dimensional uncertainty^27^, and within-dimension conjunction search^43^. In the present study, we provide evidence that this network is involved in the representation of task relevant feature dimensions in a target template using RSA. Correlations with the dimension-weighted model were observed bilaterally across the whole attention network^18^, in lateral prefrontal cortex and in occipital visual areas.

To our knowledge, this is the first paper to show that widespread regions of frontal, posterior parietal, and occipitotemporal cortices represent feature dimensions differently depending on their task relevance. Left superior parietal lobule at the border to the anterior intraparietal sulcus showed one of the highest correlations between observed RD patterns and the dimension-weighted model in the delay period (see Table 2). Previous studies have found that this area is involved in sustaining task-related signals in working memory over a delay^44, 45^ performing inefficient visual search for conjunction objects^46^, binding features into conjunctions^47^, and shifting attention between different feature dimensions presented simultaneously in a display^5, 48^. The results of our study show that anterior superior parietal lobule is additionally involved in representing task relevant feature dimensions in a target template.

Smaller clusters in frontal cortex corresponding to frontal eye fields and lateral prefrontal cortex also showed correlations with our dimension-weighted model. There is ample evidence that the frontal eye fields are involved in preparing eye movements for visual search during spatial working memory^49^, but this region has also been implicated in feature-based attention in both humans^50^ and monkeys^51^. Our finding that the frontal eye fields are involved in representing task relevant feature dimensions during the delay period suggests an additional role for this region in target template maintenance.

Lateral prefrontal cortex is a particular region linked to working memory, and its role has traditionally been seen as an attention-directing region similar to posterior parietal cortex, rather than a region that represents the contents of visual stimuli^52^. Nevertheless, recent evidence suggests lateral prefrontal cortex does code content-specific information during a delay^22^, consistent with the current findings. Finally, posterior temporal cortex has previously been found to represent the contents of complex objects in the target template^15, 53^, as well as showing high-level feature and shape-selective processing during object viewing^54–56^ and conjunction processing^57^. The current results provide evidence for a role of posterior temporal cortex in representing task relevant feature dimensions.

One question we must address concerning these results is whether the observed correlations with the dimension-weighted model were simply due to global signal strength differences between tasks. We do not think this is likely. We observed stronger univariate activation elicited by the orientation search task during the cue period, but not during the delay period. In contrast, RSA revealed more widespread correlations with the dimension-weighted model during the delay period than the cue period. This reverse temporal pattern appears to rule out a major contribution of global signal strength to the observed correlations with the dimension-weighted model.

The dimension-weighted model was represented in large parts of the frontoparietal attention network. This differed strongly from the representation of the HMAX C1 model, which modeled the similarity between sensory features as they would be coded by complex cells in visual cortex^23^. The HMAX model was mostly represented in visual cortex, as expected. In accordance with the foveal presentation of our cues, the HMAX model representation was observed in the right foveal confluence zone^58^ (confluence of foveal representations of V1, V2, and V3) around the occipital pole. The right dominance of this representation is likely to be a thresholding effect, but it is in accordance with an anatomically larger area of the right confluence zone^58^.

Unexpectedly, activation at the junction of the intermediate frontal sulcus and frontomarginal sulcus in left frontopolar cortex also correlated with the HMAX model in the cue period. Activation in this rostral frontal area was previously observed during visual dimension changes^27^. Moreover, it is likely involved in the allocation of attention resources across a broad range of paradigms ^59^. The present data show that left frontopolar cortex represents not the attended visual dimension, but stimulus features independent of the attended dimension. This fits with the hypothesis that left lateral frontopolar cortex is vital for detecting potential targets in previously unattended dimensions, thereby enabling subsequent attention changes to the previously unattended dimension^60^.

Together these results paint a picture of task relevant representations activated in various cortical regions prior to search, from cue processing to the template maintenance period. Specifically, we provide evidence that frontal, posterior parietal, lateral occipit﻿al, and early visual cortex all contribute to the representation of target feature dimensions and frontal cortex and ventral visual regions contribute to the representation of viewed features regardless of task relevance. In conclusion, top-down factors greatly influence the cortical representation of visual information during preparation for visual search.
